# Brain-Derived Neurotrophic Factor: A Key Molecule for Memory in the Healthy and the Pathological Brain

**DOI:** 10.3389/fncel.2019.00363

**Published:** 2019-08-07

**Authors:** Magdalena Miranda, Juan Facundo Morici, María Belén Zanoni, Pedro Bekinschtein

**Affiliations:** Laboratory of Memory Research and Molecular Cognition, Institute for Cognitive and Translational Neuroscience, Instituto de Neurología Cognitiva, CONICET, Universidad Favaloro, Buenos Aires, Argentina

**Keywords:** BDNF, hippocampus, perirhinal cortex, Alzheimer disease, memory, depression, stress

## Abstract

Brain Derived Neurotrophic Factor (BDNF) is a key molecule involved in plastic changes related to learning and memory. The expression of BDNF is highly regulated, and can lead to great variability in BDNF levels in healthy subjects. Changes in BDNF expression are associated with both normal and pathological aging and also psychiatric disease, in particular in structures important for memory processes such as the hippocampus and parahippocampal areas. Some interventions like exercise or antidepressant administration enhance the expression of BDNF in normal and pathological conditions. In this review, we will describe studies from rodents and humans to bring together research on how BDNF expression is regulated, how this expression changes in the pathological brain and also exciting work on how interventions known to enhance this neurotrophin could have clinical relevance. We propose that, although BDNF may not be a valid biomarker for neurodegenerative/neuropsychiatric diseases because of its disregulation common to many pathological conditions, it could be thought of as a marker that specifically relates to the occurrence and/or progression of the mnemonic symptoms that are common to many pathological conditions.

## BDNF: A Dynamically Regulated Player in Synaptic Plasticity and Memory

The brain derived neurotrophic factor (BDNF) belongs to a family of neurotrophins that have a crucial role in survival and differentiation of neuronal populations during development (Huang and Reichardt, 2001). In the adult brain, BDNF also maintains high expression levels and regulates both excitatory and inhibitory synaptic transmission and activity-dependent plasticity (Tyler et al., 2002; Wardle and Poo, 2003).

The expression of BDNF is regulated during transcription and translation, and also by post-translational modifications. The presence of a complex multi-level regulation demonstrates the importance and diversity of BDNF functions. Transcription is controlled by multiple promoters that determine activity-dependent and tissue specific expression (Timmusk et al., 1993; Chen et al., 2003). There have been identified at least four BDNF promoters in the rat (Timmusk et al., 1993), each one driving the transcription of mRNAs that contain one of the 8 non-coding exons spliced to the common 30 coding exons, which produce an heterogeneous population of BDNF transcripts. BDNF splicing has been described for several species, including humans (Liu et al., 2005), mice (Hayes et al., 1997), and rats (Timmusk et al., 1993). Additionally, the expression of specific BDNF exons can be regulated by epigenetic mechanisms (Lubin et al., 2008), suggesting that environmental experiences dynamically influence mature BDNF levels.

Regarding the pattern of expression of BDNF in the brain, high levels of this molecule have been detected in the hippocampus, amygdala, cerebellum and cerebral cortex in both rodents and humans, with the highest levels found in hippocampal neurons (Hofer et al., 1990; Timmusk et al., 1993). Lower levels of BDNF have been detected in organs such as the liver, heart, lung, among others (Ernfors et al., 1990; Maisonpierre et al., 1991). The regulation of each transcript is controlled and/or modulated by factors like neuronal activity (Metsis et al., 1993), exercise (Oliff et al., 1998), antidepressants (Russo-Neustadt et al., 2004), stress (Lauterborn et al., 1998), and hormones such as estrogens (Singh et al., 1995).

**FIGURE 1:**
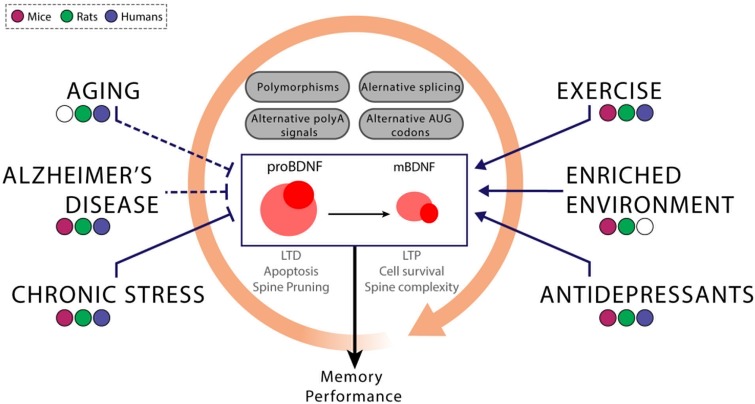
The interplay between genetic and environmental factors modulates the expression of the BDNF variants. BDNF gene expression is controlled at many levels. The inclusion of particular exons and the differential use of polyadenylation sites and/or starting codons modify not only the BDNF variant but also the temporal dynamics of its expression through the modification, for instance, of the stability of the BDNF mRNA. Variability at any of these levels of regulation can lead to differential levels of mature BDNF in healthy or diseased subjects. These differences could be related to genetic (i.e., Val66Met and other polymorphisms) or environmental factors (chronic stress, exercise, and amongst others). In addition to correlational studies performed in humans, the use of non-human animal models, particularly rodent models, can lead to establish certain relationships schematized above between these factors. Blunt arrows indicate that a decrease in BDNF expression and the sharped ones symbolize the opposite pattern. Although the consensus agrees that aging, the development of Alzheimer’s disease and the exposure to chronic stress are related to reductions in BDNF levels, some studies could not find those associations or found the opposite pattern (cases represented with dashed lines). On the other hand, some external interventions are able to enhance BDNF expression, such as exercise, enriched environment and antidepressants. For this reason, the aforementioned interventions could be potential treatments for cognitive impairments related to low BDNF expression. Since these external factors can interact with each other (represented by the orange circular arrow), it is important to take into account all of these potential interactions at the time of determining causal links between the effects of any of these variables on BDNF expression.

Brain derived neurotrophic factor is synthesized as the precursor proBDNF, that can be stored in either dendrites or axons (Lessmann et al., 2003), and undergoes cleavage intra or extracellularly (Lee et al., 2001; Mowla et al., 2001) to produce a mature BDNF protein. BDNF is released in an activity dependent manner as a mixture of pro and mature BDNF (Pang et al., 2004). Interestingly, BDNF and proBDNF are associated with opposing effects on cellular function, which gives BDNF protein function an additional level of complexity. The proBDNF form is secreted under both pathological and non-pathological conditions (Barker, 2009). ProBDNF preferentially binds p75 NTR receptor, which facilitates LTD (Woo et al., 2005) and induces apoptosis (Friedman, 2010). On the other hand, BDNF in its mature form binds specifically to tyrosine kinase receptors (TrkB) and promotes cell survival (Volosin et al., 2006), facilitates LTP and increases spine complexity (McAllister et al., 1999; Zagrebelsky et al., 2005). When p75_*NTR*_ is co-expressed with TrkB receptor it increases neurotrophins binding affinity thereby facilitating ligand discrimination (Bibel et al., 1999). In this way, proBDNF can be thought as part of a regulatory mechanism of BDNF activity in non-pathological conditions. In addition, the truncated forms of TrkB receptor can act as dominant negative inhibitors of BDNF signaling by internalizing and clearing BDNF from the synapse (Haapasalo et al., 2002; Figure 1).

Many studies have shown the critical role of BDNF for the regulation of plastic changes in the adult brain, including regulation of the trafficking (Caldeira et al., 2007), phosphorylation (Lin et al., 1998) and expression levels of NMDARs (Suen et al., 1997) associated with augmented synaptic strength. Due to its critical role in LTP, BDNF has been postulated to be an essential part of the cellular mechanism supporting memory formation and maintenance by promoting synaptic consolidation (Bramham and Messaoudi, 2005). According to this hypothesis, BDNF increases memory storage by favoring changes in spine morphology leading to the stabilization of LTP. BDNF can also increase the number, size and complexity of dendritic spines (Horch and Katz, 2002; Alonso et al., 2004), probably through unpregulated actin polymerization (Rex et al., 2007). Furthermore, BDNF increases neurogenesis through changes in cell survival (Lee et al., 2007) and proliferation (Katoh-Semba et al., 2002).

Changes in synaptic connections are thought to support memory storage. There are several lines of evidence that directly link BDNF with learning and memory. For example, BDNF could be a mediator of the plastic changes underlying both spatial and recognition memory processes (Kesslak et al., 1998; Mizuno et al., 2000; Cirulli et al., 2004; Bekinschtein et al., 2007; Heldt et al., 2007).

In this review, we will focus on the role of BDNF in cognitive function in the adult brain under normal and pathological conditions, and evaluate the potential therapeutic actions of BDNF for the treatment of cognitive alterations associated with aging, neuropsychiatric and neurodegenerative diseases. We will particularly focus on the effects of these treatments on mnemonic function.

## The BDNF Val66Met Polymorphism

In the human BDNF gene, a common single nucleotide polymorphism identified with a Met to Val substitution at codon 66 in the pro-domain of BDNF, called rs6265 or Val66Met polymorphism, affects synaptic targeting of BDNF-containing vesicles and activity-dependent neuronal release of BDNF (Egan et al., 2003). Met66 homozygous knock-in mice showed a selective impairment in activity-dependent synaptic plasticity *in vitro* (Ninan et al., 2010). Additionally, exogenous application of proBDNF in Val-carriers facilitated LTD and inhibited LTP, while not in Met carriers (Kailainathan et al., 2016). Because of its frequency in the human population [up to 30% Met carriers in a European sample (Egan et al., 2003)] and its association with lower serum levels of BDNF (Ozan et al., 2010), this single nucleotide polymorphism has been a matter of great interest. This polymorphism has been associated with structural (Pezawas et al., 2004) and functional differences in the brain, such as synaptic plasticity (Kleim et al., 2006) and memory performance (Hariri et al., 2003). Some of these structural changes include volumetric decreases in specific regions -such as the hippocampus (Szeszko et al., 2005), the parahippocampal gyrus, the prefrontal cortex and the amygdala (Matsuo et al., 2009; Montag et al., 2009)-. The presence of the Met allele is, in turn, associated with poor mnemonic performance on verbal tasks both at short and long delays and also deficits in working and spatial memory tasks (Dempster et al., 2005; Hansell et al., 2007; Goldberg et al., 2008). Since most studies relayed mainly on tasks that evaluate item memory, and particularly, verbal memory, the effects over other more complex cognitive functions remain to be tested (Mandelman and Grigorenko, 2012). Yogeetha et al. (2013) did evaluate multiple types of memories, finding only an influence on visuospatial memory, although Raz et al. (2009) describes an additional effect of associative memory.

Besides these correlational studies of Met allele dosage, hippocampal volume/activation and memory performance in healthy subjects, evidence for a role of Val66Met polymorphism in brain structure and function is not conclusive and, even meta-analysis studies show patterns of conflicting results. Kambeitz et al. (2012) conducted three separate meta-analyses to determine the effect of the Val66Met polymorphism on declarative memory performance, hippocampal volume and hippocampal activation in humans. They reported that all these measures are reduced in carriers of the Met allele compared to Val homozygotes and this effect cannot be explained by random variables such as age, gender or diagnosis criteria. However, Dodds et al. (2013) argued that the effect sizes of fMRI data are susceptible to the method used to select the voxels and that the one used in Kambeitz et al. may have led to an inflated estimate of the effect size. In fact, the meta-analysis conducted by Mandelman and Grigorenko (2012) did not detect a significant association between the BDNF Val66Met polymorphism and several phenotypes including general cognitive ability, memory, executive function, visual processing skills, and cognitive fluency. They hypothesized alternative scenarios to explain this incongruence in the literature and proposed that instead of being grouped by their behavioral similarity, cognitive phenotypes should be categorized depending on the brain activation pathways engaged. Although an important group of studies could not found an association between Val66Met genotype and memory performance (Tsai et al., 2008; Houlihan et al., 2009; Karnik et al., 2010), the results linking BDNF polymorphism and memory are not likely spurious. It is possible that differences in the effect that the BDNF gene exerts across the lifespan and the uncontrolled effects of variables such as gender, stress and physical exercise, known to affect BDNF levels, may have led to a dilution of the effect in certain samples. Furthermore, Met carriers might be able to compensate for the deficiency in BDNF levels. In fact fMRI studies suggest that they present increased medial temporal lobe activity during the engagement of an episodic memory task when compared with non-carriers, and this might “hide” the potential deficits (Dennis et al., 2011). In conclusion, these studies suggest that presence of the Met allele may confer a disadvantage in cognitive performance, and particularly episodic memory (for a review, see Bath and Lee, 2006), but that the effects of BDNF polymorphism may be too complex to be analyzed under the idea of a simple “risk allele.”

## BDNF in the Aging Brain

Aging is a major risk for the development of cognitive disorders (Horn and Cattell, 1966). Cognitive performance gradually declines with age, probably as a result of cellular and metabolic changes that lead to a progressive reduction in synaptic plasticity in brain regions crucial for cognitive functions (Barnes, 1994; Smith et al., 2000; Erickson and Barnes, 2003). Aging is related to a significant shrinkage of gray matter (Driscoll et al., 2003, 2009; Raz et al., 2005), an important reduction in the number of synapses (Burke and Barnes, 2006) and also changes in neuroplasticity-related proteins (Assuncao et al., 2010; Erickson et al., 2010). The hippocampus is a brain region with high levels of plasticity-related molecules (Neves et al., 2008) and is particularly sensitive to aging-related cellular alterations that lead to volume reductions (Greene and Naranjo, 1987; Lee et al., 1994; Rosenzweig and Barnes, 2003). Particularly numerous changes were reported in the dentate gyrus local inhibitory and excitatory circuitry (Patrylo and Williamson, 2007). Furthermore, decreases in adult neurogenesis in the dentate gyrus start emerging at middle age and continue throughout the aging process (Drapeau et al., 2003). Other structures also linked to episodic memory formation, such as the frontal and entorhinal cortices, also show volume reduction and may contribute to the deterioration of cognitive function (Driscoll et al., 2009).

At the cellular level, changes in hippocampal LTP have also been reported in aged animals, with deficits either related to induction, maintenance and/or expression of LTP depending on the stimulation pattern used for induction (Erickson and Barnes, 2003; Gooney et al., 2004). Given that alterations in the thresholds for LTP and LTD in the MTL region have been associated with impairments in long-term memories (for review, see Kumar, 2011), age-related cognitive impairment could also be linked to altered LTP function.

In fact, one of the most consistent plasticity-related deficits associated with aging is the reduction in neurotrophic signaling. BDNF-LTP in the dentate gyrus is impaired in the aged brain, and may be reverted by endogenous BDNF induction by manipulations such as ampakine administration (Rex et al., 2006), which can also ameliorate age-associated memory deficits in rodents (Granger et al., 1996). Since dysfunction of synaptic plasticity and changes in neuronal activity are associated with worse performance in different cognitive tasks (Lynch et al., 2006), efforts have been done to link changes in BDNF levels to aging-dependent cognitive decline and to the related alterations in structural and functional integrity of neuronal networks (for review, see Tapia-Arancibia et al., 2008). Mattson et al. (2004) suggested that age-dependent impairment in cognitive function could be associated with decreases in BDNF expression in the primary regions of the brain affected by aging. Consistent with this idea, the circulating concentrations of BDNF are reduced in aged primates and humans (Hayashi et al., 2001; Shimada et al., 2014) and brain concentration reduced in rats (Silhol et al., 2005). In aged rodents, the BDNF system is affected at different levels, including reduced transcription, protein synthesis and processing (Calabrese et al., 2013), however, other publications could not find an association between age-related plastic changes and BDNF (Lapchak et al., 1993; Driscoll et al., 2012). The found reductions correlate with hippocampal shrinkage (Erickson et al., 2010), spatial memory decline (von Bohlen und Halbach, 2010) and neuronal atrophy (Murer et al., 2001). For example, higher BDNF mRNA induction levels were reported after Water Maze task on unimpaired rats in comparison with aged animals (Schaaf et al., 2001). The decrease in BDNF levels observed in aged individuals, was accompanied by a reduction in the expression and/or activation of TrkB receptor and a concomitant increase in the levels of proBDNF and p75_*NTR*_, suggesting the presence of additional age-related deficits in BDNF signaling pathway and in the processing of proBDNF to mature BDNF. Additionally, these changes were negatively correlated with performance on the Water Radial Maze (Buhusi et al., 2017). Furthermore, the induction of specific BDNF transcripts after fear conditioning is altered in aged rats (Chapman et al., 2012), suggesting that aging is not only associated with reduced neurotrophin expression under resting conditions, but might lead to a functional impairment of BDNF in response to a specific task. This is consistent with the aforementioned role of BDNF in activity and experience-dependent structural and functional connectivity changes.

However, some researchers found no change or even an increase in BDNF (Lapchak et al., 1993; Narisawa-Saito and Nawa, 1996; Newton et al., 2005) associated with aging. This could suggest that the loss of hippocampal BDNF is not part of the mechanisms involved in age-related cognitive decline. However, considering that neuronal cell death is an undoubtedly important element of age-related cognitive impairment (Morrison and Hof, 1997), a transient BDNF-related response to neuronal degeneration could be the underlying reason for the increasing levels of hippocampal BDNF reported in some studies. However, since *in vivo* measurements of BDNF in the brain are not possible, studies in humans rely on inferences about the levels of BDNF on central nervous system. Given that there is evidence that BDNF can cross the blood-brain barrier (Pan et al., 1998), these studies assume that serum BDNF is a proxy of BDNF levels in the brain. Consistently, measures of BDNF in the central nervous system (brain BDNF, b-BDNF) correlate with measures of BDNF from the periphery (serum BDNF, sBDNF) (Sartorius et al., 2009; Klein et al., 2011) with as much as 75% of brain origin BDNF (Rasmussen et al., 2009). However, the functional significance of BDNF in the serum is a matter of debate. BDNF is also secreted in several peripheral sites such as platelets, lymphocytes, and skeletal and smooth muscle cells, and also recent studies have questioned the idea of BDNF being able to cross the blood-brain barrier (Pardridge et al., 1998; Di Lazzaro et al., 2007). Another important consideration in these measurements is whether pro and mature forms of BDNF are being measured, because only some assays can differentiate mature BDNF and, considering that they can have opposing effects, these should be taken into account (Polacchini et al., 2015). For this reason, the results of serum studies should be interpreted cautiously, as this could be a potential cause for inconsistencies reported in sBDNF levels between studies. The use of enriched extracellular vesicles of neuronal origin from peripheral blood could provide a novel way to bypass these issues since they could more closely reflect brain changes compared to plasma (Mustapic et al., 2017; Suire et al., 2017).

There are several reasons to think that a decrease in BDNF levels could be detrimental to normal brain functioning, like its role in synaptic plasticity, as described above. Another motive is that BDNF exerts various trophic effects on hippocampal neurons that could help counteract the noxious effects of neuronal cell death (Almeida et al., 2005). In fact, high levels of BDNF in the hippocampus are related to both survival and differentiation of dentate gyrus progenitor cells in the adult (Pencea et al., 2001; Shetty et al., 2004), and low levels of BDNF have been linked to deficient neurogenesis in aged animals (Apple et al., 2017). Moreover, BDNF is known to increase with oxidative stress as part of an antioxidant defense during aging (Mattson et al., 2002). Neuronal loss is an important component of normal aging, however, it does not contribute significantly to learning and memory impairments (West, 1993; Rapp and Gallagher, 1996; Rasmussen et al., 1996), suggesting that memory deficits associated with aging are more likely related to alterations in synaptic physiology and aberrant cell signaling, that might contribute to an altered connectivity (Lister and Barnes, 2009).

While aging-related neuroanatomical changes are evident, there is an enormous variability amongst healthy individuals in the pattern of cognitive decline (Morris and Price, 2001). Memory performance is also partly under genetic control (Payton, 2006), probably because of age-sensitivity in many cognitive processes (Kremen et al., 2007; Lessov-Schlaggar et al., 2007). Genetic variance could explain these individual differences in cognitive capacity, especially since heritability of cognitive function increases over the lifespan as brain resources decrease (Haworth et al., 2010), and can account for as much as one third of the variance in cognitive decline (Finkel et al., 2005). Both apolipoprotein E (APOE) (Wisdom et al., 2011) and BDNF (Miyajima et al., 2008) have been associated with variance in cognitive performance in healthy individuals more frequently than other genes, although some results have not been reliably replicated (Harris and Deary, 2011).

Since BDNF decreases throughout life (Erickson et al., 2010), it would be interesting to assess the possible associations between the Val/Met polymorphism and age-related cognitive decline. For example, Miyajima et al. (2008) reported poor verbal recall in a sample of healthy elderly Met homozygotes, and Sambataro et al. (2010) found that Met carriers showed greater age-related decline in hippocampal activation during both encoding and retrieval, while other studies also limited to older adults found no impact of age on cognitive tests evaluating learning and memory (Houlihan et al., 2009; Laing et al., 2012). Considering these results, this polymorphism could help understand individual differences in cognitive function by genetic-dependent changes in neurotransmitter and neurotrophic factor levels, amongst other factors (Raz and Lustig, 2014). However, since reports indicate that both APOE and BDNF polymorphisms accounts for less than 2.3% of the variance, it is fundamental to take into account the complex interplay between associations with other genes and interactions with environmental factors to interpret these results. Many environmental and hormonal factors such as physical exercise (Cotman and Berchtold, 2007), caloric restriction (Mattson et al., 2003), estrogen levels (Scharfman and Maclusky, 2005), and environmental enrichment (van Praag et al., 2000) can influence BDNF levels, making it challenging to link BDNF to age-related memory impairment and hippocampal atrophy. For example, BDNF genotype can modulate the effect of physical exercise on episodic memory performance and brain volume. This is evident by the fact that only Val homozygous benefited from physical exercise with larger MTL volume and hippocampal gray matter, whereas in Met carriers the contrary effect was found (Brown et al., 2014). In another study, in a cohort aged 65 or older the strength of the association between incidence of cognitive decline and physical activity increased with the number of Met alleles, suggesting that the Met allele may confer vulnerability to dementia in elders with less physical activity (Kim et al., 2011). On the other hand, there are studies reporting a reduced vulnerability of Met carriers to age-related decline in executive function (Harrisberger et al., 2014), pointing toward a differential effect of BDNF on cognitive function related to the areas supporting the task.

The influence of BDNF on cognitive function may change across the lifespan. In fact, the effects of the BDNF Val66Met polymorphism on brain structure and cognitive function were found to differ in an age-dependent manner: while Met carriers showed a reduction in episodic memory performance and hippocampal/parahippocampal volume in samples of around 65 years in comparison to Val carriers (Egan et al., 2003; Pezawas et al., 2004), in the elderly (mainly samples of around 75 years) Val/Val individuals had diminished entorhinal cortex thickness, white matter tract integrity, and episodic memory performance (Harris et al., 2006; Erickson et al., 2008; Voineskos et al., 2011). It has been hypothesized that this effect could be related to changes in the level of cleavage, since cleavage molecules such as tPA are known to decline with age (Cacquevel et al., 2007) which could create a paradoxical effect where greater BDNF secretion would, in fact, lead to cognitive decline (Pang et al., 2004). Another explanation could be a decrease in the penetrance of the BDNF genotype across the lifespan, as other factors such as the independent incidence of age-related diseases increase their influence on brain structure and cognition (Lindenberger et al., 2008). In fact, there are results that suggest that Met carriers have more preserved frontostratial functions than Val/Val subjects (Gajewski et al., 2012), what may lead to an increase in the use of stratium-dependent mnemonic strategies, that could give a potential advantage on Met carriers that might hinder the original deficits reported in young individuals. This might help explain conflicting results in this regard, since there are studies showing that aged Met-carriers still have deficits in mnemonic performance and they experience a steeper impairment in memory tasks as they age (Kennedy et al., 2015) and have diminished performance in remembering neutral faces when compared with Val/Val individuals (Mascetti et al., 2013).

Aging is normally accompanied by a loss of memory function (Erickson and Barnes, 2003). Episodic memories are particularly more sensitive to the aging process (Verhaeghen et al., 1993) than procedural or non-declarative memories (Light, 1991). Within the limits of the episodic memory domain, some aspects can be more vulnerable to aging than others. For example, associative memory tasks that require binding of multiple pieces of information can be more sensitive to the aging process (Naveh-Benjamin, 2000; Old and Naveh-Benjamin, 2008). This deficit is mostly related to spatial (Tanila et al., 1997; Oler and Markus, 1998) and recognition memory loss (Moss et al., 1988; Danckert and Craik, 2013). The vulnerability of particular mnemonic processes to aging is probably due each of these functions being supported by distinct brain regions showing differential rates of functional decline with age (Buckner, 2004). Diminished input from the EC to the DG could contribute to deprive the HP from sensory information crucial for discrimination of novel versus familiar stimuli when this stimuli are similar (Wilson et al., 2005; Holden and Gilbert, 2012), and in fact reductions in DG-CA3 connectivity are associated with spatial learning decline in old animals (Smith et al., 2000). The medial temporal lobe region (MTL), that is thought to support episodic memory function, is particularly vulnerable to cellular alterations that happen during aging and/or pathological dysfunction (Jobst et al., 1994). Since episodic memory decline is correlated to decreases in hippocampal volume (Charlton et al., 2010), these changes could explain age related mnemonic deficits. Another important structure for episodic memory in the MTL is the perirhinal cortex (Prh), a region involved in the discrimination of novel and familiar stimuli (Malkova et al., 2001; McTighe et al., 2010) that is particularly crucial to solve tasks involving ambiguous features (Bartko et al., 2007). Aging alters discrimination in both in rats and humans increasing a propensity to identify novel stimuli as familiar (Plancher et al., 2009; Burke et al., 2010). This effect was, in some cases, interpreted as a deficit in the ability to bind features of an object, so that decisions are made based on the familiarity of a single component. The consequence is the incapacity to detect novel compositions of familiar features (Jones and Jacoby, 2005). In fact, complexity and ambiguity of the features was proposed as a determining variable in recognition memory deficits in aged rats (Burke et al., 2011; Gamiz and Gallo, 2012), as well as in Prh-lesioned animals using a configural task with complex objects (Norman and Eacott, 2004). Several molecular and biochemical alterations have been reported in the Prh of aged animals that could contribute to the associated cognitive deficits (Liu et al., 2008; Moyer et al., 2011). Since exposure to novel objects is related to an increase in BDNF levels in the Prh (Romero-Granados et al., 2010), and familiarity discrimination in the presence of ambiguous stimuli (and not clearly distinguishable ones) is impaired with BDNF antisense ODNs infusions during a restricted time window after the task (Seoane et al., 2011; Miranda et al., 2017), BDNF is an interesting molecular candidate that could help to establish a link between molecular and biochemical alterations and the pattern of both spatial and recognition memory deficits associated with aging. In particular, because optimal cognitive function is linked to efficient neuronal plasticity, these memory deficits might be coupled to alterations in the expression and regulation of plasticity-related proteins such as BDNF, a protein whose expression is both affected in the aging brain and is crucial for memory consolidation and particularly for discrimination of similar memories.

In correspondence with this idea, decrease in BDNF expression has been associated with neuronal atrophy and death occurring in some neurological disorders (Murer et al., 2001). Administration of exogenous BDNF can prevent pathological changes in the nervous system associated with aging (Nagahara et al., 2009) [but see Fischer et al. (1994) for inconsistent results; for review, Fumagalli et al. (2006)], and can rescue both BDNF-induced LTP and spatial memory performance in aged animals (Rex et al., 2006). Since BDNF has been linked to synaptic plasticity, neurogenesis, neuronal survival and protection against brain insults (Bath and Lee, 2006), the above results imply the possibility that BDNF could act as a synaptic repair molecule. There are a few evidence that support this idea, for example, acute application of the TrkB agonist 7,8-dihydroxyflavone rescues synaptic plasticity in the hippocampus of aged rats *in vitro* (Zeng et al., 2011). Additionally, chronic treatment also prevents age-related impairments in contextual and cued fear conditioning with a simultaneous normalization of the spine levels that normally decrease with age (Zeng et al., 2012). Furthermore, the Lou/C rat, an animal model of successful aging that presents a preserved cognitive performance across its longer lifespan (Kollen et al., 2010), showed higher hippocampal BDNF than Wistar rats and a decrease in proBDNF with age. This contrasts with the increase in proBDNF seen in aged Wistar rats (Tapia-Arancibia et al., 2008). However, the beneficial effect of BDNF on neuroprotection and mnemonic performance in rats decrease as age increases (Sohrabji and Bake, 2006), probably due to additional changes in the processing and signaling pathway. Consistent with the role of BDNF on synaptic plasticity and memory, elderly Lou/C rats never showed short- or long-term memory decline in recognition memory tasks or impaired LTP (Kollen et al., 2010). However, Silhol et al. (2007) found that learning-associated cognitive training could increase TrkB receptor expression in aged animals and also increased proBDNF processing both in aged and young rats, indicating that learning leads to a strengthening of BDNF pathway, especially in aged animals where this pathway is affected.

## BDNF and Alzheimer’s Disease

Reduced levels of BDNF have been reported not only under normal aging conditions but also in pathological conditions including Huntington (HT), Alzheimer’s disease (AD), and Parkinson’s disease. However, the profile of cognitive deficits greatly differs between these pathologies according to the brain regions affected by degeneration. For example, the most profound BDNF deficits are reported in the hippocampus, parietal, entorhinal and frontal cortex for AD (Hock et al., 2000) and in the striatum and motor cortex for HT (Zuccato et al., 2008). In this section we will focus on AD because it starts mainly as impairment in declarative memories, without affecting other neurological functions (Walsh and Selkoe, 2004). It has been proposed that this feature is related to the degenerative profile of the disease that starts in the hippocampus, parahipocampal cortices and amygdala, but not in primary sensory and motor cortices (Selkoe, 2001).

There is a substantial amount of studies supporting the idea that neurotrophic factors are crucial for the etiology of AD, in particular BDNF. BDNF protein and mRNA levels (Hock et al., 2000) as well as proBDNF (Peng et al., 2005) are reduced in the post-mortem brain of AD patients compared with age-matched controls, with no changes in TrkB levels (Savaskan et al., 2000). This reduction was also reported in Mild Cognitive Impairment (MCI) (Shimada et al., 2014), a potentially prodromal stage of AD (Flicker et al., 1991). Furthermore, reduced circulating levels of BDNF were also found in MCI (Forlenza et al., 2010). BDNF levels are correlated to the severity of the disease and with episodic memory performance in patients (Peng et al., 2005), suggesting that these decreases could be related to the pathogenesis of the disease. In conclusion, downregulation of BDNF and proBDNF are thought to be an underlying mechanism related to early AD (Peng et al., 2005). However, Laske et al. (2006) found that patients in the early stages of AD had significantly higher sBDNF levels than patients in the late stages and also than age-matched controls. This highlights that it is difficult to establish a causal link between BNDF downregulation and the development of this neurodegenerative disease because the pathology is accompanied by a loss of cell density and dendritic spines that could secondarily affect BDNF levels. In this regard, there are also post-mortem and serum level studies that report an increase in BDNF and TrkB concentrations in the hippocampus and parietal cortex of AD patients (Durany et al., 2000; O’Bryant et al., 2009). This increase may be related to compensatory mechanisms that could contribute to the repair by degradation of β-amyloid. In addition, other potential moderators could contribute to differences and heterogeneity seen in these studies. Differences in diagnostic criteria, stages of the disease, sex and education and the use of pharmacological treatments such as acetylcholinesterase inhibitors or psychotropic medication that are known to raise BDNF levels (Leyhe et al., 2008), or could come from other potential sources outside the CNS such as immune cells (Kerschensteiner et al., 1999).

Given that synaptic loss is the major correlate of cognitive impairment, much stronger than the presence of plaques or tangles (Terry et al., 1991), there is a recent view of AD as a “synaptic pathology” (Lippa et al., 1992; Heffernan et al., 1998). Aβ monomers are normally generated and secreted at firing synapses, and are not toxic but neuroprotective as they have an active role in synaptic regulation (Giuffrida et al., 2009) and are crucial for neuronal function (Abramov et al., 2009). Aβ monomers are one of the many factors that regulate synaptic function and they can activate CREB via the PI3K/AKT pathway, leading to a sustained CREB-regulated transcription and release of BDNF (Giuffrida et al., 2018; Zimbone et al., 2018). In this way, BDNF can act as a converging point of many synaptic regulators. In Alzheimer’s disease (AD), neurotoxic β-amyloid (Aβ) oligomers are formed from the self-association of Aβ monomers. These oligomers can promote neurotoxicity through different ways (Pearson-Leary and McNay, 2012). Arshavsky (2006) suggested that the selective vulnerability of memory related areas could be, in fact, a result of specific cellular modifications required for the process of memory consolidation. An important event in AD is the pathogenic Aβ-mediated alterations in the levels of neurotrophic factors (NTFs) (Budni et al., 2015). Since pathogenic Aβ oligomers cannot can activate PI3K/AKT pathway and induce CREB activation, the increase in the levels of Aβ-oligomers can lead to an impairment in CREB activation in the brain of patients with AD and mouse models of AD (Bartolotti et al., 2016). Soluble Aβ oligomers are known to alter signal transduction pathways crucial for learning and memory processes such as CREB-regulated transcription (Caccamo et al., 2010) and trafficking of NMDA type of glutamate receptors (Snyder et al., 2005). Thus, alterations in those pathways could play an important role in the etiology of the disease. Altered levels of BDNF in AD are downstream of Aβ-accumulation and could be related to Aβ–induced dysregulation of CREB transcription (Caccamo et al., 2010; Pugazhenthi et al., 2011). Even if BDNF does not modify Aβ accumulation, it could have an important function in moderating the effects of Aβ on cognitive and structural aspects (Nagahara et al., 2009). BDNF protects against Aβ-mediated toxicity by contributing to its degradation and preventing tau hyperphosphorylation (Elliott et al., 2005; Tapia-Arancibia et al., 2008). In this sense, BDNF is expressed by microglial and astroglial cells in the plaque vicinity and seems to protect from neuroinflammation, thereby supporting neuronal survival (Lindvall et al., 1994; Kerschensteiner et al., 1999) and preventing apoptosis (Tamatani et al., 1998). On the other side, Aβ down-regulates BDNF mRNA *in vitro* via reduction of CREB (Rosa and Fahnestock, 2015) and disrupts retrograde axonal transport of BDNF (Poon et al., 2011) and conversion of pro-BDNF to mature BDNF (Zheng et al., 2010). It also interferes with synaptic plasticity mediated by BDNF even at concentrations that do not kill the cells (Wang et al., 2006). This downregulation occurs before the appearance of plaques and is linked to memory deficits in AD animal models (Francis et al., 2012) and in MCI (Peng et al., 2005). Tau, a mediator of Aβ-induced toxicity, can significantly downregulate BDNF via transcript IV both *in vitro* and *in vivo* by itself (Rosa et al., 2016). As mentioned before, many studies found that decreases in serum BDNF levels can be detected in individuals with MCI, so it is tempting to speculate that BDNF loss could be involved as an early event in this synaptic dysfunctions. However, the presence of some inconsistencies between studies with MCI patients warns us to be cautious with these speculations. Nevertheless, these results suggest a critical role of BDNF in the regulation of Aβ-amyloid toxicity, suggesting that BDNF dysregulation could contribute to synaptic dysfunction and mnemonic impairment related to AD. This data implies that, although central to the development of AD, changes in BDNF expression could be an effect of earlier functional modifications in other synaptic related proteins. In particular, one of these proteins could be Aβ, that in its monomeric form has a normal physiological role in synaptic plasticity and neuronal survival in the brain and can actually have an active role in these BDNF changes by regulating BDNF transcription and release (Parihar and Brewer, 2010). In any case, the beneficial effects of BDNF on memory and cognition could reflect its synapse repair features.

Changes in the cell microenvironment, where a lack of trophic support can lead to a decrease in neuronal survival and proliferative activity (Drapeau and Nora Abrous, 2008), could contribute to the degeneration of specific neuronal subpopulations in pathological conditions. During this period, changes in BDNF levels contribute to age-related hippocampal volume changes, and atrophy associated with pathological conditions (Erickson et al., 2012). There is evidence of as much as 1–2% annual hippocampal atrophy in the elderly without signs of dementia, while in patients with AD this deterioration goes up to 3–5% per year (Jack et al., 1998). In patients with MCI, hippocampal volume is predictive of rapid conversion to dementia (Jack et al., 1998), evidencing its importance in the progress of the disease.

Most studies report that BDNF genotype is not related to the risk of developing AD (Combarros et al., 2004; Nishimura et al., 2004; Li et al., 2005) [but see for evidences of effects present only in women (Fukumoto et al., 2010)], and Genome Wide Association Studies could not find a relationship between BDNF Val66Met polymorphism and risk of AD (Lambert et al., 2013). However, some studies do report an increase in the risk for AD in Val carriers (Ventriglia et al., 2002; Matsushita et al., 2005; Voineskos et al., 2011). Other studies found an association between Met carriers and greater rates of decline in episodic memory and hippocampal atrophy in patients with MCI (Forlenza et al., 2010; Lim et al., 2013, 2016), leaving Aβ accumulation unaffected (Lim et al., 2013). Although there are certain inconsistencies among the literature, BDNF role in the development of AD seen with Val66Met has been replicated with other BDNF polymorphisms (Kunugi et al., 2001; Riemenschneider et al., 2002). The lack of consistency between studies could be related to differential effects of BDNF during distinct stages of the disease, with more circulating BDNF in MCI patients, and less in AD patients (Yu et al., 2008; Forlenza et al., 2010). Lower BDNF levels may be linked to neuronal death in AD, concealing any effect of the BDNF gene. Since the complexity of the pathological changes stemming from the disease increases as the severity progresses, associations between BDNF Val66Met polymorphism and AD should be more obvious in preclinical stages in which the disease presents almost exclusively subtle alterations in mnemonic performance (Fahnestock, 2011).

Neurotrophic factors not only moderate neuronal and synaptic dysfunction but also cognitive decline in AD (Fahnestock, 2011). Higher sBDNF is associated with a protection against future occurrence of dementia and AD (Weinstein et al., 2014) and predictive of slower rates of decline (Laske et al., 2011). In the same manner, changes in BDNF levels induced pharmacologically or by aerobic exercise are related to better cognitive function and diminished synaptic dysfunction both in humans at risk of developing AD and in animal models of AD (Baker et al., 2010; Intlekofer and Cotman, 2013). These effects could be related to the ability of BDNF to prevent lesion-induced neuronal degeneration (Morse et al., 1993; Kiprianova et al., 1999). According to this idea, post-lesion gene transfer of BDNF partially restored the deficits in learning capacity and synaptic plasticity in an AD model in which BoNTx-induced damage to the entorhinal cortex was used to mimic AD pathology (Ando et al., 2002). Neural stem cell transplants or CREB binding protein gene transfers reversed spatial memory deficit via BDNF in AD mouse models, despite widespread Aβ plaque and tau pathology (Blurton-Jones et al., 2009; Caccamo et al., 2010). In a recent study, delivery of BDNF to the entorhinal cortex in amyloid transgenic mice reversed neuronal atrophy and synaptic loss, regulated neuronal signaling, and diminished the related mnemonic deficits without changes in the amyloid plaque load (Nagahara et al., 2009) indicating that BDNF can act through amyloid-independent mechanisms to exert its protective effect. Furthermore, 7,8-dihydroflavone (7,8-DHF), Neotrofin (a hypoxanthine derivative that stimulates neurotrophic factor production) and Neuropep-1 (a BDNF modulating peptide) have shown to reverse memory deficits in animal models of AD or even in preclinical trials (Glasky et al., 1994; Devi and Ohno, 2012; Shin et al., 2014). In this way, BDNF could mediate the protective effect of exercise and caloric restriction on neurodegeneration (Vaynman et al., 2004b). This strengthens the need to develop behavioral interventions that could prevent the risk of developing dementia or slow the progression to dementia in patients with MCI, a path that is currently in progress. Many of these new paths point to lifestyle changes that range from antioxidant diet, environmental enrichment and social interaction to physical or cognitive exercise as potential interventions (Fahnestock et al., 2012).

## The Effect of Chronic Stress on BDNF and the Link to Psychiatric Disorders

Chronic stress is a known factor involved in the incidence of AD and cognitive impairment (Wilson et al., 2007c). Structures involved in the control of the physiological status of an organism are susceptible to modulation by chronic stress. In particular, the hippocampus is altered by prolonged exposure to aversive situations (Kim et al., 2015). These abnormalities are reflected in deficits in spatial memory tasks and novel object recognition (Luine et al., 1994; Vedhara et al., 2000; Baker and Kim, 2002), but also in altered synaptic plasticity processes (Shors et al., 1989; Kim and Yoon, 1998) like suppression of LTP (Artola et al., 2006). Chronic stress typically decreases BDNF hippocampal expression (Smith et al., 1995; Murakami et al., 2005), however, when the cause of the stress disappears, the hippocampus shows amelioration of the cognitive and synaptic deficits (Sousa et al., 2000; Hoffman et al., 2011).

To this date, a wide variety of strategies have been assessed to reduce the deleterious effects caused by chronic stress. Infusions of BDNF in the rat hippocampus before a chronic restraint stress protocol can protect against the deficits in learning and memory in the MWM and in LTP (Radecki et al., 2005) and shRNA against BDNF before a stress protocol can revert the spatial reference memory deficits during the post-stress-rest period.

Exercise, is a well-known strategy to increase BDNF brain levels, so it has been proposed as a non-invasive way to mimic the effects of direct BDNF administration over chronic stress. Radahmadi et al. (2016) found that the hippocampal BDNF increases in response to exercise after a chronic stress protocol. On the other hand, Dief et al. (2015) showed that animals that followed a 30 days swimming training program improved their performance in the T maze after being exposed to chronic stress and this enhancement correlated with upregulation of hippocampal BDNF. Also, Kwon et al. (2013) described a BDNF-mediated improvement in MWM performance in chronically stressed mice that started treadmill running 12 weeks before the beginning of the stress protocol and continued throughout it.

Shafia et al. (2017) investigated the palliative effects of exercise (alone or combined with fluoxetine) on a rat model of post-traumatic stress disorder. This model shows impairments in fear conditioning and extinction, inhibitory avoidance task and location recognition memory. Interestingly, in most tests, the effects of the combined treatment were similar to the ones obtained with exercise alone. They also found, in agreement with the work of Garza et al. (2004), that exercise alone and exercise plus antidepressant enhanced hippocampal BDNF expression, but not antidepressant alone.

Enriched environment (EE) has been shown to increase BDNF levels in the hippocampus in comparison with standard housing conditions (Novkovic et al., 2015). Thus, EE could be an easy way to promote the systemic and neural recovery from the effects of chronic stress. Shilpa et al. (2017) showed that exposure to EE following 10 days of immobilization (2 h/day) ameliorates spatial memory deficits in a version of the radial arm maze and depressive-like behavior. Recovery seems to be achieved through the modulation of several signaling cascades, including BDNF’s.

Seong et al. (2018) suggested that EE is as effective as Fluoxetine when it occurs after exposure of the animals to a chronic stress protocol, but additional measures of the effectiveness of the stress protocol would be needed to establish the success of the treatment. Interestingly, BDNF levels were increased in the hippocampus of rats that receive either EE or Fluoxetine in comparison with the control group (stressed but without posterior treatment). Considering that chronic stress is linked to depressive-like symptoms (Garcia, 2002; Calabrese et al., 2009), the results obtained using antidepressants do not seem surprising. The mechanisms of antidepressant actions over chronic stress and the putative involvement of BDNF have also been extensively studied but with no consistent results yet. Larsen et al. (2010) showed that chronic antidepressant treatment reversed depressive-like behavior caused by chronic unpredictable stress-induced and increased BDNF mRNA expression in the granular cell layer of the dorsal hippocampus (independently of exposure to stressors). Using a different stress model, Tsankova et al. (2006) were able to normalize behavioral alterations in mice exposed to a social defeat stress protocol followed by chronic (but not acute) administration of imipramine They proposed a model in which chronic stress induces repression and chronic imipramine induces de-repression of the *bdnf* gene in the hippocampus through changes in the chromatin structure.

Some studies are focusing in compounds that have been originally used to treat other diseases but have shown some antidepressant effects in animal models, such as resveratrol. Resveratrol and curcumin, when chronically administrated, prevent the behavioral and biochemical alterations induced by chronic restraint and unpredictable stress, respectively, and those effects seem to be mediated by an increment in the expression of BDNF (Xu et al., 2006; Zhang et al., 2017). Zhou et al. (2017) show that biperiden alleviates depression-like symptoms induced by chronic unpredictable stress, increasing performance in the sucrose preference, novelty suppression feeding and forced swimming tests. Importantly, these effects were inhibited by pretreatment with the TrkB antagonist K252a.

Since the evidence suggests that BDNF may drive the recovery from stress-induced effects on the hippocampus, an interesting question emerges: Is BDNF capable of reversing the effects of chronic stress in the presence of the stressor?

Radahmadi et al. (2016) tested the effect of exercise during the exposure to stressors (“protective exercise”). Unlike preventive and therapeutic exercise, no increment on BDNF hippocampal levels was found. On the other hand, Miller et al. (2018) explored, in mice, the potential palliative effects of running on chronic stress-related impairments when exercise and stress are co-occurring. They found that the TrkB receptor had higher expression levels in both exercised groups (stressed and non-stressed) compared with both sedentary groups, supporting the hypothesis that the mitigation of the negative consequences of stress by exercise could be mediated by BDNF.

Depending on the chronic stress protocol (duration and type of stressor), different and even contrasting results have been found (Vasquez et al., 2014). BDNF appears to be an important underlying molecule behind the restitution of a normal cognitive phenotype in animal models of chronic stress. The fact that BDNF could be increased with non-invasive protocols and/or drugs -some of which are used in clinical trials- makes it attractive for human therapies.

The link between stress, specific genes and the development of psychiatric disorders has been extensively studied (Abbott et al., 2018), and a causal role has been ascribed of gene-environment interactions in the etiology of many of them (Rogers et al., 2019). In fact, psychiatric disorders can be defined as clinical entities emerging from the genetic-environmental interaction (for review, Gallo et al., 2018).

## Psychiatric Disorders and BDNF

In the last few years, evidence from animal models and clinical studies strongly suggest that dysregulation of neurotrophic factors could play an important role in the etiology of the bipolar disorder (BD), major depressive disorder (MDD), and schizophrenia (SZ) (Duman and Monteggia, 2006; Autry and Monteggia, 2012; Nieto et al., 2013). Due to the role of BDNF in neural plasticity, there could be a link between BDNF expression and the cognitive symptoms associated with memory impairments (Autry and Monteggia, 2012).

The *mnemonic* domain is commonly affected in different psychiatric disorders, such as BD (Zhou et al., 2018; Lin et al., 2019), MDD (Roca et al., 2015; Ahern and Semkovska, 2017), or SZ (Ricarte et al., 2017). Moreover, studies of post-mortem brain tissue of patients with BD and MDD reported that BDNF levels are decreased in structures involved in memory processes, such as the hippocampus (Reinhart et al., 2015) and the prefrontal cortex (Dwivedi et al., 2003). In the case of SZ, post-mortem brain tissue analyses have shown more controversial results. While some studies observed an increase in BDNF expression in the prefrontal cortex (Takahashi et al., 2000) and the hippocampus (Iritani et al., 2003), others have shown a decrease in both structures (Weickert et al., 2003; Issa et al., 2010). Although BDNF was originally thought as a viable indicator of pathological brain functioning for early detection of BD, MDD, SZ, or AD, the discriminative power of BDNF as a biomarker is highly limited, since it seems to be a non-specific marker of many neuropsychiatric disorders.

The BD is a neuropsychiatric disorder that emerges from the interaction between genetic and environmental factors and is characterized by the switching between maniac and depressive episodes (for review, Harrison et al., 2018). It has been proposed that BDNF signaling participates in the physiological effects produced by some pharmacological treatments used for BD (Shaltiel et al., 2007). It has been shown that sBDNF decreases in the first episode of unmedicated BD patients and that, after 1-year of pharmacological intervention, sBDNF concentration increases (Palomino et al., 2006). in addition, there was a a negative correlation between the number of episodes and sBDNF levels (Kauer-Sant’Anna et al., 2009). It has been reported that sBDNF positively correlates with the duration of the maniac and depressive episodes (Dias et al., 2009). This evidence suggests that episode-related changes in the structure of the brain could be linked to peripheral BDNF concentration. Cao et al. (2016) have shown that hippocampal volume is reduced in patients with BD that present Val66Met BDNF polymorphism compared with controls and patients with MDD. Moreover, they proposed a link between the hippocampal volume and the performance in an episodic memory task. Another work has shown that the peripheral BDNF correlates with the performance in episodic memory task in BD patients with the BDNF Val66Met polymorphism (Chang et al., 2018). In this line, a recent study has shown that high levels of sBDNF are associated with good cognitive performance, including verbal memory (Mora et al., 2019). This evidence suggests that changes in BDNF expression in BD patients could produce structural modifications in the hippocampal formation related to episodic memory impairments. Despite this, most of the studies report alterations in other cognitive domains that are important for a good performance in episodic memory tasks, such as attention and working memory (for review, Sole et al., 2018). Thus, BDNF dysregulations could be related with the emergence of more complex symptom’s profiles. For this reason, the relationship between BDNF and episodic memory in BD remains unclear.

Major depressive disorder is one of the most common mood disorders worldwide and is characterized by the absence of pursuit of pleasurable activities and the presence of negative thoughts (Kim and Moore, 2019). Since most common drugs used as antidepressant block the serotonin transporter (SERT), increasing extracellular serotonin in the raphe’s nucleus post-synapses (for review, Teissier et al., 2017), it has been proposed that a misbalance in the serotonergic release could be related to the etiology of the depressive symptoms (for review, Liu et al., 2018). BDNF regulates the growth and reconstruction of 5-HT containing neuronal terminals in the cortex (Mamounas et al., 1995), and administration of BDNF in the raphe nucleus reduces behaviors related to depressive symptoms in rats (Siuciak et al., 1997). In addition, MDD patients present cognitive decline in different domains (Zuckerman et al., 2018), including episodic memory (Jayaweera et al., 2016) but only recently these deficits have been studied in detail. A large amount of work shows that sBDNF is decreased in MDD (Molendijk et al., 2011). Oral et al. (2012) found that patients that recurrently present depressive episodes show lower levels of sBDNF compared with those patients that were cursing their first episode. Interestingly, antidepressant treatment increases sBDNF concentration (Molendijk et al., 2011), but there is no consensus on whether lower sBDNF correlate with poor performance in memory tasks observed in this pathology (Oral et al., 2012).

In the case of the SZ, different studies have shown that the level of sBDNF correlates with cognitive performance in different domains (Carlino et al., 2011). Despite the lack of consensus on whether basal sBDNF is increased or decreased in SZ patients (Fernandes et al., 2015), some studies have indicated a correlation between memory performance and sBDNF levels (Zhang et al., 2012; Hori et al., 2017). Interestingly, there are evidences that pro-cognitive effects of pharmacological interventions in SZ could be mediated by BDNF (Einoch et al., 2017). For example, Zhang et al. (2018) have found that a 12-week chronic treatment with olanzapine produced an increase in BDNF plasma concentration. Moreover, BDNF concentration positively correlated with cognitive performance in a RBANS scale of memory. Not only the pharmacological interventions were effective on the reduction of mnemonic symptoms, different cognitive training protocols were also designed to enhance specific cognitive domains, especially memory (Guimond et al., 2018; O’Reilly et al., 2019). Fisher et al. (2016) conducted a computerized cognitive training in SZ patients and the patients exposed to this program present higher levels of sBDNF compared to the control group. They observed an enhancement in memory, but a causal link between sBDNF and memory remains unclear (Heitz et al., 2018).

## BDNF as a Potential Mediator Underlying the Benefits of Therapeutic Strategies

Considering all the results mentioned above, it would be tempting to suggest the use of BDNF as a therapeutic target for both age-related and neuropsychiatric-related cognitive dysfunction. This idea has found many difficulties to be put to practice because of the poor brain barrier penetration of BDNF and short half-life on plasma. Moreover, gene therapy and BDNF mimetic strategies came across many negative side effects that led to their abandonment (Thoenen and Sendtner, 2002). Clinically plausible alternative approaches could include a natural increase the production of endogenous BDNF (Balkowiec and Katz, 2000). In this sense, epidemiological studies have suggested that a number of lifestyle factors such as physical exercise, diet and social activity and education may reduce the long-term risk of cognitive impairment and dementia (Larson et al., 2006; Wilson et al., 2007b; Verburgh, 2015), and animal studies are consistent with this idea (Adlard et al., 2005).

In particular, the risk of developing AD is highly increased in a lonely person (Wilson et al., 2007a), indicating that social interaction could delay the onset of the disease. Physical activity is another lifestyle factor that could influence the progress of the disease. Recent reports from both epidemiological and interventional studies reinforce the idea of using physical activity as a strategy to increase neuroplasticity in pathological conditions (Gregory et al., 2012). The influence of behaviors such as exercise and social interaction on learning and memory processes has been thoroughly studied. Researchers have found a relationship between frequent social activity and improved cognitive function (Stern, 2006). In the same direction, the cognitive improvement due to physical exercise has also been well documented (Smith et al., 2010). Physical exercise has shown not only to ameliorate structural changes in the brain, but also to protect against aging-related cognitive decline (Voss et al., 2013; Duzel et al., 2016).

Considering that lifestyle implementations have the ability to impact in the brain, a central question is how these changes in energy metabolism and social stimuli can impact on the brain structure and interact with synaptic plasticity and molecular systems to improve cognitive function.

A current model explains the effects of these lifestyle factors in terms of changes in vasculature and neurotrophic and neurotransmitter-support system (Vivar et al., 2013). Of all these changes, BDNF is the only one that is present in all the aforementioned lifestyle manipulations. BDNF is increased by social interaction with conspecifics in APP/PS1 mice, leading to the reversal of memory deficits (Hsiao et al., 2014). Also, BDNF could be important for the regulation of energy homeostasis, since diminished BDNF levels are associated with disorders of energy metabolism such as obesity and hyperglycemia (Rios et al., 2001). In fact, a high fat diet was shown to decrease BDNF levels in the hippocampus and impair learning and memory (Molteni et al., 2002a). Additionally, the increase in BDNF is one of the most consistent changes reported following exercise, as recently discussed in a meta-review (Szuhany et al., 2015). The most robust experiments supporting the fundamental role of BDNF in exercise-induced improvement in cognitive function are the ones in which blockade of BDNF impaired the cognitive improvements induced by exercise (Vaynman et al., 2004a; Garcia-Mesa et al., 2014; Kim and Leem, 2016). Vaynman et al. (2004a) showed that the exercise-induced enhancement of learning in the MWM task was blocked by TrkB-IgG administrated during the exercise period. Furthermore, exercise enabled the acquisition of sub threshold experiences (object location memory task) and this effect was dependent on BDNF. A similar effect was reported by Intlekofer and Cotman (2013) using BDNF siRNA to diminish BDNF function.

For its practicality, physical activity is the lifestyle change with more potential as a therapeutic/prevention strategy. A bulk of studies have focused on the idea of aerobic exercise as a potential non-pharmacological and low cost treatment to maintain and improve neurocognitive function (Hillman et al., 2008). A meta-analysis of several longitudinal training studies showed that exercise improved cognitive function regardless of the task type (Colcombe and Kramer, 2003). Recent studies confirmed this effect showing that not only spatial or contextual hippocampal dependent- memory tasks improve with exercise (Albeck et al., 2006; Luo et al., 2007), but also non-spatial memories such as object recognition that are thought to rely more heavily on the Prh than the HP (Hopkins and Bucci, 2010). A single session of cardiovascular exercise benefits long-term memory but does not influence short-term memory (Roig et al., 2013). Moreover, exercise can improve memory in aged animals specifically during a restricted time window after the experience, reinforcing the specific role of exercise over the memory consolidation process (Snigdha et al., 2014). Interestingly, many studies found that sex may be an important variable when evaluating the effectiveness of exercise interventions (Barha et al., 2017) and this is consistent with the sex-specific mechanisms of action of BDNF (Chan and Ye, 2017). The timing of the intervention could also be relevant. In several models of traumatic neurological injury, when interventions are given prior to the damage, induction of BDNF reduced neuronal degeneration and improved cognitive outcome (Bruce-Keller et al., 1999; Zhang et al., 2011). Although the effects of exercise are somewhat short-lived (Alaei et al., 2007; Hopkins and Bucci, 2010), some interventions can improve the outcome even when given after the damage (Griesbach et al., 2004). However, the duration of the benefits depends on the age of the subject during the exercise exposure. While adolescent exercise training did not affect BDNF levels immediately after an object recognition task, it did lead to greater BDNF levels in the Prh if the task was done 2 weeks after. In adulthood, exercise increased BDNF levels immediately after the task but this effect was short-lived and lasted less than 2 weeks (Hopkins et al., 2011). These data suggest that exercise could modulate learning related plastic changes in an age-dependent manner. Although the benefits of exercise are related to many growth factors, BDNF is the only one consistently elevated after a few weeks of continuous exercise (Molteni et al., 2002b). This neurotrophin is rapidly induced in the hippocampus and cortical regions (Cotman and Berchtold, 2002), and remains elevated for several days after exercise (Erickson et al., 2010). In addition, BDNF levels can be rapidly re-induced up to peak levels by a subsequent sub threshold exposure to exercise, even several days after the end of the exercise program (Berchtold et al., 2005).

Exercise does not influence brain regions uniformly, but affects them in a more selective way, which suggests location-specificity of the molecular pathways involved in exercise-induced plasticity. Interestingly, the effects of exercise on BDNF expression occur in regions related to mnemonic functions such as the anterior hippocampus, cerebellum and frontal cortex, but not others such as the striatum (Neeper et al., 1996). This is in accordance with previous reports indicating that exercise-induced increases in sBDNF levels are associated with changes in hippocampal volume, which, in turn, correlate with spatial memory performance (Erickson et al., 2011).

The positive effects of exercise on plasticity are particularly relevant for the aging population, in which BDNF levels are decreased (Erickson et al., 2010). Considering that the aging brain is still capable of plasticity, lifestyle related experiences could be a way to recruit plastic processes and counteract the detrimental effects of aging (Churchill et al., 2002). In aged animals, hippocampal neurogenesis and BDNF levels can increase with exercise (Marlatt et al., 2012). Although these effects are not as robust as those seen in younger animals (van Praag et al., 2005), the increase in BDNF seems to ameliorate mental deterioration and improve memory function (Erickson et al., 2012). In fact, long-term exercise programs are able to rescue these cognitive deficits even after the first signs of mnemonic impairment (Tsai et al., 2018). A recent clinical trial examined the impact of aerobic cardiorespiratory training versus stretching on MCI patients and reported sex-dependent cognitive improvements related with trophic factor and Ab-40 and Ab-42 circulating levels (Baker et al., 2010).

Physical activity is also associated with a lower risk of developing dementia (Friedland et al., 2001). Many clinical trials point toward improved cognition and reduced incidence of psychiatric symptoms when patients with mild AD received a physical training protocol (Hoffmann et al., 2016; Cammisuli et al., 2018). However, some studies suggest that environmental enrichment could be more beneficial for cognition than physical exercise alone (Wolf et al., 2006; Cracchiolo et al., 2007).

Coordinative exercise (Niemann et al., 2014) and cognitive training (Basak et al., 2008; Hall et al., 2009) can also induce gray matter plasticity and enhance cognitive functions in older adults. However, the improvement generated by prior training is usually domain-restrictive and only acts over memory systems affected by the previous experience (Markowska and Savonenko, 2002; Green and Bavelier, 2008). Nonetheless, there are some reports that show a generalized benefit of prior experience to different tasks and contexts (Buschkuehl et al., 2008). A combination of physical and cognitive training with control of nutritional and cardiovascular risk factors during a 2-year period led to improved cognitive performance in old adults at risk of developing dementia (Ngandu et al., 2015). In animal studies, environmental enrichment could be seen as a multidomain intervention. It consists of social enrichment, physical exercise and environmental changes and has been shown to increase BDNF levels and enhance learning and memory in different domains such as object recognition, spatial learning and motor abilities (Greenough et al., 1972; Frick and Benoit, 2010). The combination of both sensory enrichment and physical activity has more impact on neuronal plasticity than these elements given independently. This motivated original therapeutical proposals in human research. For example, a novel dancing program with higher cognitive and coordinative demands than previous physical activity programs induced more gray matter increases in an aged group than a traditional sport program with comparable cardiovascular demands (Muller et al., 2017; Rehfeld et al., 2018).

One important question regarding the effects of exercise on cognitive function is to establish the mechanisms responsible for the cross talk between cardiovascular/muscle activity and the central nervous system. The muscle higher metabolic rates could lead to the secretion of signaling molecules, that could subsequently upregulate plasticity related gene expression and protect the brain from damage. In this way, elevated plasticity molecules such as BDNF could prime the brain to be better prepared for subsequent changes related to learning or could be selectively secreted in an activity-dependent manner during learning experiences. As a result, exercise could enhance the activity of a general molecular machinery important for learning and memory. In accordance with this view, molecules such as CREB, NMDARs subunits and BDNF are particularly induced following exercise (Molteni et al., 2002b) and brain regions important for memory formation such as the hippocampus are selectively influenced by physical activity (Vaynman et al., 2004b).

A potential mechanism for the exercise-related neuroprotective effects of BDNF is via modulation of synaptic and structural plasticity. Plastic changes induced by exercise include increased neurogenesis (van Praag et al., 1999b; Merkley et al., 2014), greater arborization of neuronal dendrites and synaptogenesis (Eadie et al., 2005; Dietrich et al., 2008), as well as increased amplitude and reduced threshold for LTP (van Praag et al., 1999a). Since these effects are accompanied by a concurrent increase in BDNF levels (Ding et al., 2006; Ferris et al., 2007), BDNF could be a potential mediator. In addition, increased vascularization (Morland et al., 2017) accompanied by greater dendritic complexity and neurogenesis could explain the increase in hippocampal volume following exercise (Erickson et al., 2011). The progressive age-related decline in neurogenesis has been associated with a non-permissive microenvironment with low levels of neurogenesis-promoting factors. However, this microenvironment is still responsive to environmental changes and can be stimulated even at late stages to provide molecular cues for proliferation (van Praag et al., 2005; Kronenberg et al., 2006; Lugert et al., 2010; Silva-Vargas et al., 2013; Smith et al., 2018). Changes in growth factor levels such as BDNF might underlie the decrease in neurogenesis seen as a consequence of disease or aging, and the aged brain retains the capacity to respond to the neurogenesis-stimulating effects of growth factors.

Exogenous application of BDNF can restore the levels of hippocampal neurogenesis in aged animals (Scharfman and Maclusky, 2005). In the same way, exercise-induced increases in neurogenesis are necessary for the physical activity-dependent enhancement in learning and memory (Clark et al., 2008). This led to the idea that neurogenesis could be the substrate of this cognitive enhancements mediated by BDNF (Bekinschtein et al., 2011).

Another potential beneficial effect of BDNF is its ability to protect neurons from oxidative damage or excitotoxic stress (Cheng and Mattson, 1994; Wu et al., 2004) and from Aβ-induced degeneration (Counts and Mufson, 2010) in animal models of normal and pathological aging. In fact, BDNF is upregulated in response to different kinds of insults to the nervous system (Hsu et al., 1993; Yang et al., 1996; Hayashi et al., 2000). An exercise regime can lessen the accumulation of oxidative cell damage and the dysfunction characteristic of aged animals (Radak et al., 2001), and selective suppression of BDNF increases the vulnerability of neurons to excitotoxicity (Jiang et al., 2005) and increases amyloidogenesis (Matrone et al., 2008).

Although there should be a clear excitement to establish a systematic exercise program to ameliorate or even prevent symptoms of memory deficits related to aging, psychiatric disorders or diseases, many limitations still exist for this therapeutic line. One of the most important drawbacks of this approach is that the high prevalence of chronic diseases in the aged population affects exercise performance and feasibility, increasing the potential risks of the treatment, especially for high intensity protocols (Hundley et al., 2001). This directly impacts on the motivation to follow these kinds of treatments, which are known to have low adherence (Kosse et al., 2013). Programs with lower intensities could be a better choice, since they are still able to impact positively on cognition and neurophysiology in aged subjects without affecting adherence to the treatment (van der Bij et al., 2002). It has been reported that the lack of time is also one of the main reasons for avoiding regular practice of aerobic exercise (Gillen and Gibala, 2014). Thus, there is interest in developing more efficient training programs involving less time demand but inducing a similar BDNF response. Pietrelli et al. (2018) demonstrated, in animals, that the practice of low/moderate intensity aerobic exercise through 2 to 18 months of life increased BDNF in various brain structures like the prefrontal cortex and the hippocampus and reduces the normal decline due aging. Moreover, this protocol improved both novel object recognition and context discrimination capacity. Szuhany et al. (2015) conducted a meta-analysis to determine the impact of acute and regular exercise on BDNF levels in humans. They found that the moderate effect from a single session of exercise was intensified if it was executed after a regular program of exercise. They posit that each episode of exercise results in a “dose” of BDNF activity and that the magnitude of this “dose” can be enhanced over time by regular exercise.

On the other side, attempts to directly use recombinant BDNF as a therapy have found many methodological limitations. One of the main problems for translating BDNF-based therapies into the clinic is problem of delivery to the brain and the challenge of sustaining the expression for longer intervals since since the recombinant protein has a very short half-life. In this sense, some preclinical studies were oriented to using BDNF fused to cell-penetrating peptides and packed in AAV-constructs and intranasal delivery of these AAV constructs to central nervous system (Arregui et al., 2011; Ma et al., 2016). A10-day-AAV treatment could alleviate depression-like behaviors in mice (Ma et al., 2016), and AAV delivery of BDNF striatal neurons induced neurogenesis and increased the lifespan of an animal model of HD (Benraiss et al., 2012). Although beneficial, the use of AAV has been limited by difficulties in biodistribution and the immunogenicity to the virus. An alternative approach is the use of stem cell transplants that can express BDNF and other beneficial factors and can migrate into damaged areas of the brain by their selective tropism to inflammation and apoptosis sites, although they are not permanently integrated into the organism (Kidd et al., 2009; Joyce et al., 2010; Fink et al., 2015; Deng et al., 2016; Pollock et al., 2016). Mesenchymal stem cell transplantations have shown improvements in behavioral deficits in a murine model of HD, have also slowed the neurodegenerative processes by a diminished atrophy and an increased neurogenesis (Dey et al., 2010; Benraiss et al., 2013; Pollock et al., 2016).

An alternative possibility to the use of exercise as a ‘natural’ and non-invasive way of increasing BDNF signaling in neuronal networks, is the administration of drugs already available for clinical use (Stranahan et al., 2009). Many medications are capable of impacting on BDNF levels. Memantine and donepezil are a pharmaceutical compound used to alleviate the symptoms of AD that markedly increases BDNF levels in a dose-dependent manner (Marvanova et al., 2001; Leyhe et al., 2008; Meisner et al., 2008).

Ampakines are good candidates because they can increase excitatory transmission affecting BDNF levels. In rat hippocampal slice cultures, a very brief clinically tested ampakine treatment produced elevated BDNF protein levels that lasted several days after the exposure (Lauterborn et al., 2003). Another therapeutic possibility that has emerged recently is the use of transcranial magnetic stimulation (TMS) to increase BDNF levels. TMS restored the levels of BDNF and TrkB that were normally reduced in aged mice and improved spatial memory (Zhang et al., 2015).

In conclusion, several environmental and lifestyle interventions that reduce age-dependent cognitive decline and pathological degeneration can also increase BDNF production, suggesting that BDNF is neuroprotective (Figure 1). Given that cognitive training is a focused approach that selectively acts on sets of memory domains and that drugs are invasive, exercise is still a ‘favorite’ when thinking of potential therapeutic approaches.

## Conclusion

Although BDNF is a key player in synaptic plasticity and memory process, its role in the etiology of cognitive symptoms in pathological conditions remains unclear. BDNF-mediated plastic changes have been proposed as one of the underlying neurobiological substrates of memory consolidation, and changes in BDNF were shown to directly affect memory performance in animal models of neurodegenerative/neuropsychiatric diseases and in normal conditions. In human post-mortem brain tissue, BDNF expression is higher in memory related structures, such as the hippocampus and the amygdala. However, in humans, the relationship between memory and BDNF still remains correlational, mainly because the available techniques do not allow the control of BDNF expression in humans. Despite this difficulty, numerous studies attempted to establish causal relationships between these two factors by analyzing memory performance under conditions that up-regulate or down-regulate BDNF expression. Since brain BDNF expression correlates with sBDNF concentration, this association has been extensively used to study the implication of BDNF in mnemonic functions in humans under normal and pathological conditions. In fact, the concentration of this blood-measurable protein is correlated with the memory impairment in different disorders. Additionally, changes in the trafficking and release of BDNF due to Val66Met polymorphism have also been used to correlate BDNF levels with mnemonic performance and structural changes in memory-related regions in healthy and diseased individuals. It has been shown that interventions such as exercise, chronic administration of fluoxetine, and cognitive training can enhance the sBDNF concentration and correlates with a better performance in memory tasks. The initial idea of using BDNF as a biomarker for neurodegenerative/neuropsychiatric diseases was discarded because changes in BDNF levels are common to many pathological conditions, which underscores its discriminative value and potency. However, considering the data reviewed here, we suggest that BDNF can be thought of as a marker that specifically relates to the occurrence and/or progression of the mnemonic symptoms that are common to many pathological conditions that share deficits in this cognitive domain. Moreover, BDNF was shown to be a shared factor in which converge most of the therapies that have been able to fight these mnemonic symptoms.

## Author Contributions

MM was responsible for drafting the manuscript and revising its content. JM and MZ contributed to writing the manuscript and figure design. PB was responsible for the general idea, writing and critically revising, and correcting the manuscript. All authors read and approved the final manuscript, and contributed to the conception of the work.

## Conflict of Interest Statement

The authors declare that the research was conducted in the absence of any commercial or financial relationships that could be construed as a potential conflict of interest.
